# Breast Cancer Estimate Modeling via PDE Thermal Analysis Algorithms

**DOI:** 10.3390/bioengineering5040098

**Published:** 2018-11-05

**Authors:** Young Hoon Park, Sung Mo Yang

**Affiliations:** Department of Precision Mechanical Engineering, Chonbuk National University, 567 Baekje-daero, Jeonjusi 561-756, Korea; yangsm@jbnu.ac.kr

**Keywords:** breast cancer, diagnosis, partial differential equation (PDE), thermal analysis

## Abstract

The significance of this study lies in the importance of (1) nondestructive testing in defect studies and (2) securing the reliability of breast cancer prediction through thermal analysis in nondestructive testing. Most nondestructive tests have negative effects on the human body. Moreover, the precision and accuracy of such tests are poor. This study analyzes these drawbacks and increases the reliability of such methods. A theoretical model was constructed, by which simulated inner breast tissue was observed in a nondestructive way through thermal analysis, and the presence and extent of simulated breast cancer were estimated based on the thermal observations. Herein, we studied the medical diagnosis of breast cancer by creating a theoretical environment that simulated breast cancer in a real-world setting; the model used two-dimensional modeling and partial differential equation (PDE) thermal analysis. Our theoretical analysis, based on partial differential equations, allowed us to demonstrate that non-wounding defect detection is possible and, in many ways, preferable. The main contribution of this paper lies in studying long-term estimates. In addition, the model in this study can be extended to predict breast cancer through pure heat and can also be used for various other cancer and tumor analyses in the human body.

## 1. Introduction

The ability to detect the presence of internal defects without destroying the defective specimen, while also determining the size and position of the defects, offers many benefits, including increased time efficiency. Nondestructive research (NDT/NDE) has continued to diversify due to the emergence of infrared thermal imaging equipment. However, breast cancer research via thermal analysis is still in its early stages [1,2]. In particular, breast cancer research through partial differential equation (PDE) modeling of thermal analysis has only recently started being explored. Internal status estimation through analytic modeling has been studied by several researchers; recently, estimation of both the position and size of breast cancer tumors was accomplished by Amri et al. [3]. The original technology used to determine the presence of breast cancer through PDE thermal analysis was developed in previous research [4,5,6]. Recently, defects in stainless steel (STS) were compared using both experiment and theory via thermal image technology and PDE thermal analysis modules [7]. Further, various thermal imaging techniques have been applied in many different fields [8,9,10].

The present study built a temperature distribution model, using data from the surface of a test model, to observe the position and size of changes in simulated breast cancer. In particular, a two-dimensional model for PDE thermal analysis was used to set the thermal transmission and calculate the temperature distribution on the surface of the model, as well as to analyze temperature differences along the surface of the test model according to changes in position and size of defects. It is well known that the temperature inside a test model is increased by heat itself, and this heat is spread across the surface when passing through a defect in the test model, thus showing a higher temperature than that of the ordinary surface [11,12,13,14,15,16]. This study overcomes the limitations of animal tests by developing a new thermal analysis test. In general, animal tests are a last resort in this field, because they require the specimen to be scratched and the product is a wound. The greater the number of wound tests, the lower the economic benefits for the company and the environment. It is also generally accepted that nondestructive testing has a detrimental effect on the human body. In addition, nondestructive testing that is harmless to the human body has been unreliable and inaccurate. This study overcomes the disadvantages described above, making it possible to predict internal cancer through pure thermal analysis. This method will be useful as a new alternative that overcomes the limitations of existing nondestructive tests.

In this study, we estimated the presence, position, and size of defects through two-dimensional modeling, using a PDE theory analysis module for the purpose of studying the relationships between surface temperature, the test model, and defects.

The basis of the theoretical thermal analysis used in this study was J. P. Holman’s work on heat transfer, and the thermal analysis was performed by modeling semi-circular breast specimens in two dimensions. In this model, the cancer was classified into three categories, and the cancer location was changed from 45 degrees to 90 degrees starting from the horizontal direction. Two-dimensional thermal analysis of a specimen can be applied to various different models depending on the boundary conditions, including the specimen’s temperature, ambient temperature, heating time, specimen fluid velocity, specific heat, and density. In addition, if thermal analysis is applied after changing the modeling shape, the human body beyond the structural material range can be analyzed. The reason for choosing a semicircular model is that it is easy to apply such a model to the human body, and not many studies have analyzed breast materials to date. The most important contribution of this study is that PDE thermal analysis methods can predict breast cancer in a short time by using pure heat, which is harmless to the human body. We are confident that our comparative analysis using a PDE thermal analysis algorithm will be applied in various different fields, including the medical field [3]. This study has not yet conducted clinical trials due to the high cost and procedural difficulties of coordinating such clinical trials in Korea. In addition, such analysis was previously verified through theoretical and experimental studies with metal [3,7] and the first verifications of the patent [4,5,6].

## 2. Test Materials and Methods

### 2.1. Theoretical PDE Thermal Analysis

The partial differential equation (PDE) analytical, numerical calculation used in this study adopted a physical model to analyze the evaluation procedure [4,5,6].

If *q* is the heat transfer coefficient, $\frac{\partial T}{\partial x}$ is the heat temperature gradient, *k* is the coefficient of thermal conductivity, and $\dot{q}$, *c*, and *ρ* are the generated energy per unit volume, specific heat of a material, and density, respectively, then they form a general equation in three dimensions representing heat conduction:

(1) $$\;\frac{\partial }{\partial x}\left(k\frac{\partial T}{\partial x}\right)+\frac{\partial }{\partial y}\left(k\frac{\partial T}{\partial y}\right)+\frac{\partial }{\partial z}\left(k\frac{\partial T}{\partial z}\right)+\dot{q}=\rho c\frac{\partial T}{\partial \tau }\;$$

If the coefficient of thermal conductivity *k* is a constant, we can simplify this calculation:

(2) $$\;\frac{\partial^{2}T}{\partial x^{2}}+\frac{\partial^{2}T}{\partial y^{2}}+\frac{\partial^{2}T}{\partial z^{2}}+\frac{\dot{q}}{k}=\frac{1}{\alpha }\frac{\partial T}{\partial \tau }\;$$

Eliminating the *z*-axis and the internally generated energy $\dot{q}$(≅0) to enable the two-dimensional analysis in Equation (1) leads to the equation below [3]

(3) $$\;\frac{\partial }{\partial x}\left(k\frac{\partial T}{\partial x}\right)+\frac{\partial }{\partial y}\left(k\frac{\partial T}{\partial y}\right)=\rho c\frac{\partial T}{\partial \tau }\;$$

Converting Equation (2) into a circle through two-dimensional analysis results in the following equation:

(4) $$\;\frac{1}{r}\;\frac{\partial^{2}}{\partial r^{2}}\left(rT\right)+\frac{1}{r^{2}sin\theta }\;\frac{\partial }{\partial \theta }\left(sin\theta \frac{\partial T}{\partial \theta }\right)+\frac{\dot{q}}{k}=\frac{1}{\alpha }\frac{\partial T}{\partial \tau }\;$$

Equation (4) was used as the partial differential thermal analysis equation for the circle modeling; this is the first time that this equation has been studied in depth, although some partial results can be found in the literature [1,2,3,4,5,6,7].

In addition, we assume the boundary conditions of the thermal equilibrium equation are

(5) $$\;-k\frac{\partial T}{\partial x}=\;hf\;\left(Ts-Tf\right)+\sigma \varepsilon \left(Ts^{4}-Tf^{4}\right)\;$$

where *hf* is the surrounding heat convection coefficient, *Ts* is the material surface temperature, *Tf* is the surrounding air temperature, *σ* is the Stefan–Boltzmann constant, and *ε* is the radiation constant of the material surface.

**Figure 1:**
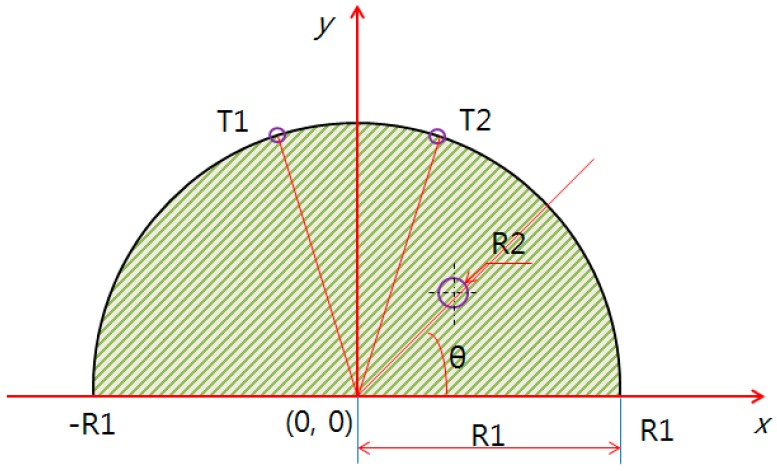
Modeling of the evaluation diagnosis.

Figure 1 shows the thermal analysis model used in this study, where R1 indicates the left and right coordinates of both endpoints of the test model, the defect is located at R2, and the surrounding temperatures are analyzed at T1 and T2.

### 2.2. PDE Analysis and Breast Cancer Research Flow

The purpose of this study is to (1) create a fast and easy way of determining the presence, position, and size of breast cancer using the temperature difference and size of the material surface by applying a two-dimensional breast model, and (2) analyze how fast and accurate the cancer–tumor evaluation is over long-term timescales.

**Figure 2:**
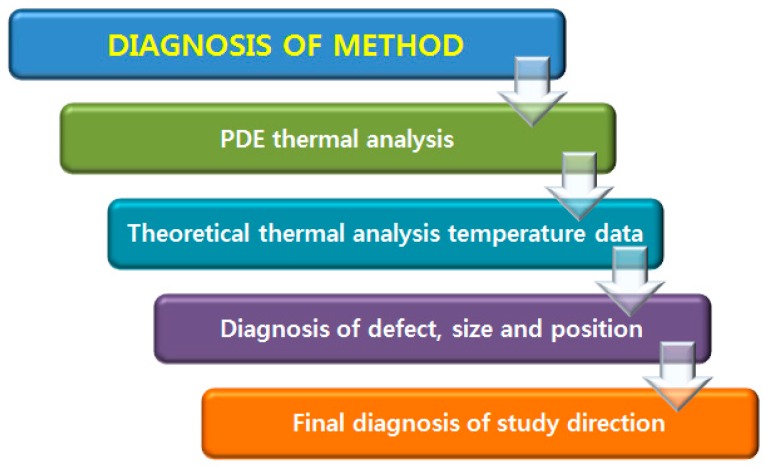
Flow chart of the study. PDE: partial differential equation.

The validity of this study was verified via a model analysis of tumor diagnosis [4,5]. Furthermore, internal STS and duralumin defects have also been estimated using two-dimensional modeling thermal analysis, validating the accuracy of these methods [2]. Figure 2 shows the research flow of the comparative data analysis.

The thermal analysis in this study was used to numerically analyze the PDE according to its mathematical properties and boundary conditions. We have reformulated Equation (3) according to the analytic modeling conditions [3]:

(6) $$\;\rho c\frac{\partial T}{\partial t}=\nabla k\;\nabla T-\omega_{b}\rho_{b}C_{b}\left(T-T_{a}\right)\;$$

where *T* is the temperature of the breast model, *t* is the time, *k* is the thermal conductivity of the breast tissue, *T_a_* is the surrounding temperature, *ω_b_* is the blood fluid velocity inside the breast model, *ρ_b_* is the density of the breast model, and *C_b_* is the specific heat inside the breast model. In addition, the defects were evaluated and the thermal analysis model was constructed using the boundary condition in Equation (5).

The model establishes theoretical data using the surface temperature difference as a function of the size and position of the breast cancer. This is accomplished through PDE analysis of the surface temperature distribution of the breast test model based on the presence, size, and position of the breast cancer. In this model, the breast cancer (R2) position and size according to the test model radius (R1) were varied. Equations (6) and (7) were used as the governing equation and boundary conditions, respectively [1]. Equation (7) changes Equation (5) to breast cancer experimental conditions:

(7) $$\;-k\frac{\partial T}{\partial x}=\;hf\;\left(Ts-Tf\right)+\sigma \varepsilon \left(Ts^{4}-Tf^{4}\right)\;$$

where *hf* is the surrounding heat convection coefficient, *Ts* is the test model surface temperature, *Tf* is the surrounding air temperature, *σ* is the Stefan-Boltzmann constant, and *ε* is the test model surface radiation constant.

The heat conduction equation was applied in this study by modeling the test model as a semicircle (two-dimensional). The temperature distribution, according to the breast cancer position and size, changed with the test model radius. Equation (4) was applied to the two-dimensional governing equation used for the study. Here, *r* is the radius of the breast test model and the breast cancer, *T* is the temperature, *k* is the conductivity, *𝑞̇* is the generated heat volume inside the breast cancer, *α* is the thermal diffusion coefficient, and *τ* is the time [17]. The conditions applied to this study are k = 0.48 W/m, Ts(T) = 309.5 °k, Tf(Ta) = 293 °k, $\omega_{b}$= 0.0001 s, ρb = 1100 kg/m^3^, hf = 15 W/m^2^, $C_{b}$ = 3300 J/kgK, σ = 5.69 × 10^−8^, ε = 0.98, and $\dot{q}$ = 0, *r*(R1) = 60, 70, 80 mm [3].

### 2.3. Modeling 1 (X = 10, Y = 0)

**Figure 3:**
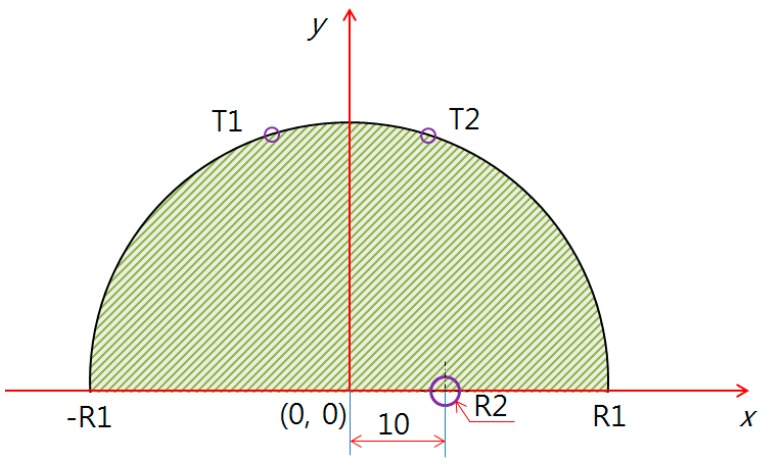
Defect position along the right-hand direction (X = 10, Y = 0).

**Figure 4:**
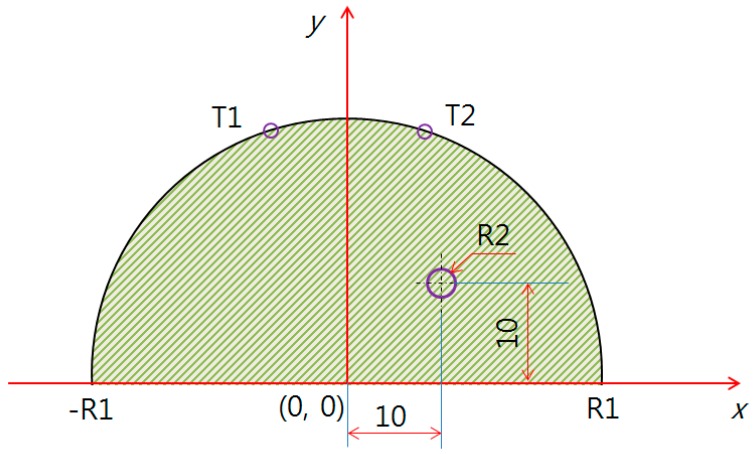
Defect position along the diagonal direction (X = 10, Y = 10).

**Figure 5:**
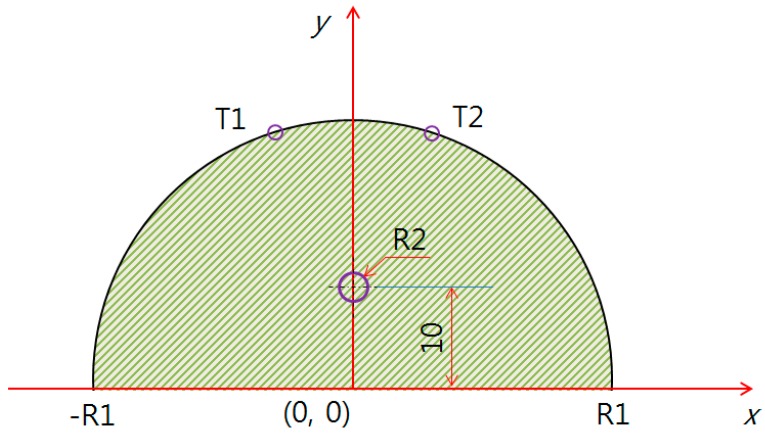
Defect position along the upper direction (X = 0, Y = 10).

The model using PDE analysis is shown in Figure 3, Figure 4 and Figure 5. The test model was given a semicircle shape in two dimensions, and the defect evaluation, based on the surface temperature difference, can be seen in Figure 5. Figure 3 shows the test model as a semicircle, where the breast cancer was a circle centered at (10, 0). The test model (R1) had a diameter whose left and right endpoints were (−R1, 0) and (R1, 0), varied by 40, 50, 60, 70, and 80 mm. To analyze the relationship between R2 (breast cancer) and the surface temperature difference, only 60, 70, and 80 mm were used, based on the possibilities of breast cancer research. Furthermore, we only used a positive coordinate due to the *y*-axis symmetry.

### 2.4. Modeling 2 (X = 10, Y = 10) and 3 (X = 0, Y = 10)

Figure 4 shows the middle of the breast cancer at (10, 10), whereas Figure 5 shows (0, 10).

The shape of the breast cancer at R2 was a circle, and the size changed from 1 to 10 mm in integer increments. Regarding the thermal flow, the generated heat from the defect was given as thermal conduction in the test model surface, which was radiated via the external temperature of the air beyond the surface. The position and size of the breast cancer had varying temperatures according to the test model position; this was because the internally generated heat was spread across the surface as it passed through the breast cancer. The breast cancer size estimation could be calculated to below a decimal point through PDE analysis, but the analysis was rounded up to integer values due to visibility limitations and the fact that the size was below 10 mm.

## 3. Results and Discussion

### 3.1. Application of PDE Thermal Analysis

**Figure 6:**
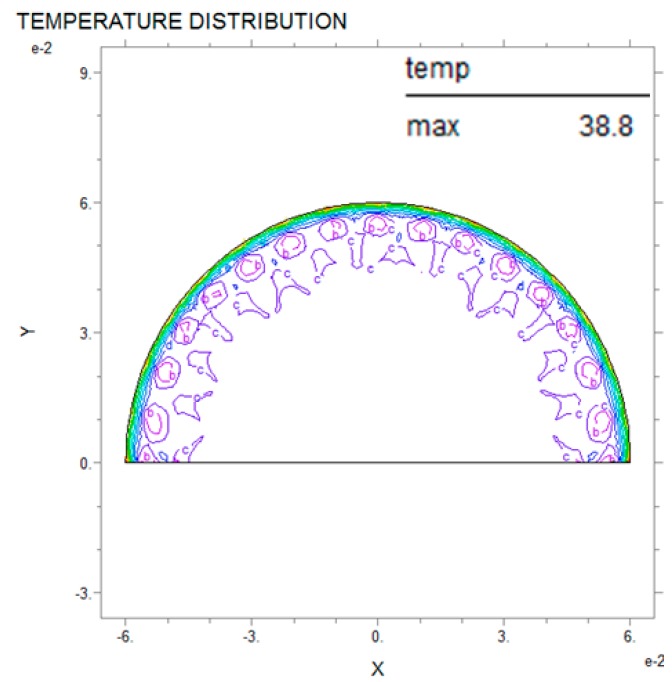
PDE thermal analysis at R1 = 60 mm, R2 = 3 mm.

**Figure 7:**
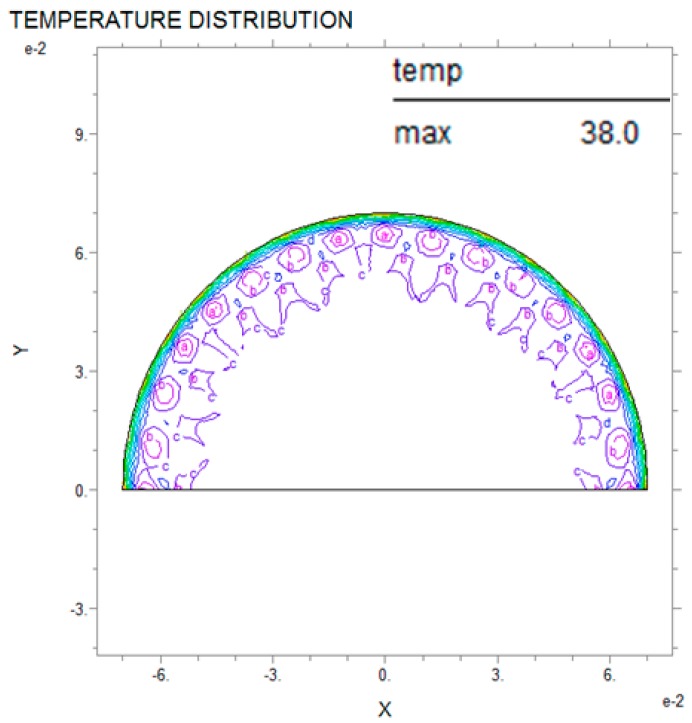
PDE thermal analysis at R1 = 70 mm, R2 = 3 mm.

**Figure 8:**
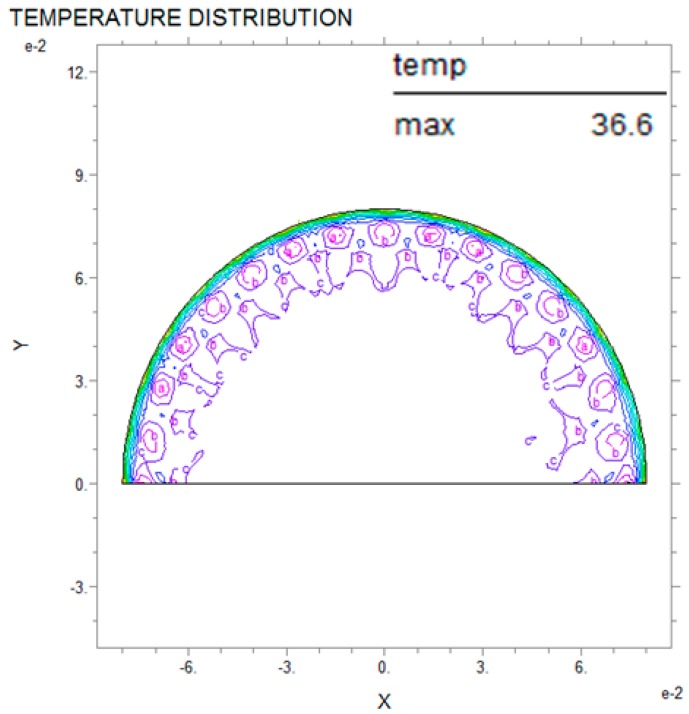
PDE thermal analysis at R1 = 80 mm, R2 = 3 mm.

Thermal analysis via partial differential equations can be used to predict breast cancer and reduce damage to the human body while also improving the precision of the diagnosis compared with existing nondestructive testing [3]. The PDE thermal analysis was performed by analyzing the entire interior of the semicircle after applying internal thermal diffusion via the boundary conditions. Figure 6, Figure 7 and Figure 8 show the PDE thermal analysis data via cross-sectional thermal analysis photographs. Although the original resolution is low, the contour line shows the thermal analysis according to the temperature. Thus, the PDE analysis of this study only used the difference between the maximum temperature and the surface temperature. The breast cancer was located on the *Y*-axis (X = 0, Y = 10). The breast cancer (R2) stayed the same and only the size of the test model (R1) was changed; this was intended to mimic how breast cancer can appear in many locations. Figure 6 shows that the relationship between R1 and R2 was 60:3 mm, and in Figure 7 and Figure 8 the relationship was 70:3 mm and 80:3 mm, respectively. The analysis results showed that the larger the R1, the slower the heat transfer and the lower the temperature distribution. The relationship between R1 and the maximum temperature was 60 mm and 38.8 °C, 70 mm and 38.0 °C, and 80 mm and 36.6 °C, and the temperature decreased more rapidly as R1 increased in increments of 10 mm. In addition, the larger the R1, the smaller the surface temperature difference. However, it was difficult to distinguish the complete temperature array from the color on the screen. Thus, the thermal image temperature distribution was quantified using the analysis tool and shown in a table. As a result of the thermal analysis, the surface temperature distribution was varied according to the breast cancer and breast size. This study aimed to predict the location and size of the breast cancer according to the breast surface temperature. If the defect and material data are scaled up as in the above results, then in the future this model could be applied to various different fields [18,19,20].

### 3.2. Relationship of R1 (Breast Test Model) and R2 (Breast Cancer)

Table 1, Table 2 and Table 3 show the temperature differences of the breast cancer position and size for the PDE analysis in Figure 3, Figure 4 and Figure 5. These tables show the defect (R2) position for (X = 10, Y = 0), (X = 10, Y = 10), and (X = 0, Y = 10), depending on the radius (R1) and the temperature difference of the surface, including the maximum and minimum values as the size changed. For example, in Table 1, if the breast test model size (R1) was 50 mm, the surface temperature difference was 0.60 °C, and the breast cancer (R2) was expected to be 3 mm. In Table 2, if the test model size was 60 mm, the surface temperature difference was 0.54 °C, and the breast cancer (R2) was expected to be 4 mm. In Table 3, if the test model size was 70 mm, the surface temperature difference was 0.55 °C, and the breast cancer (R2) was expected to be 6 mm.

In addition, it would be possible to apply such a model to various different scenarios, if the surrounding environment variables were converted to different scales, including diagnosing various types of cancers or tumors in the human body or defects in metal [21,22].

### 3.3. Relationship of R2 (Breast Cancer) and the Surface Temperature Difference

**Figure 9:**
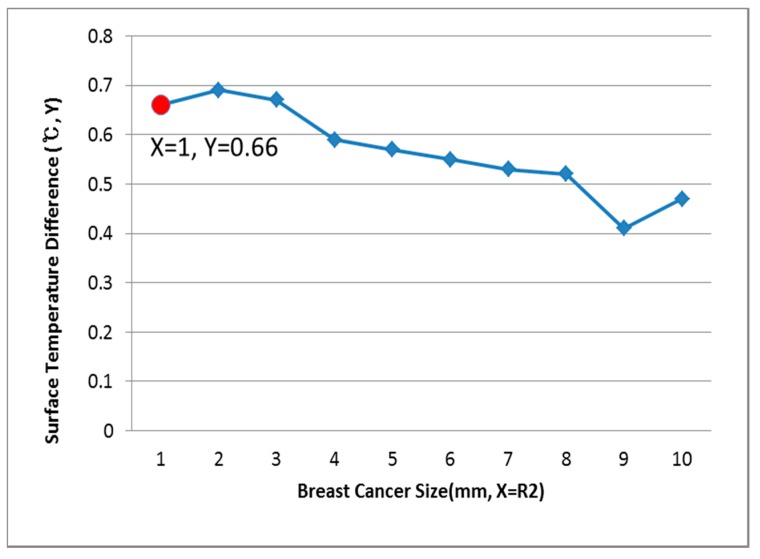
Surface temperature difference for R1 of 60 mm (X = 10, Y = 0).

**Figure 10:**
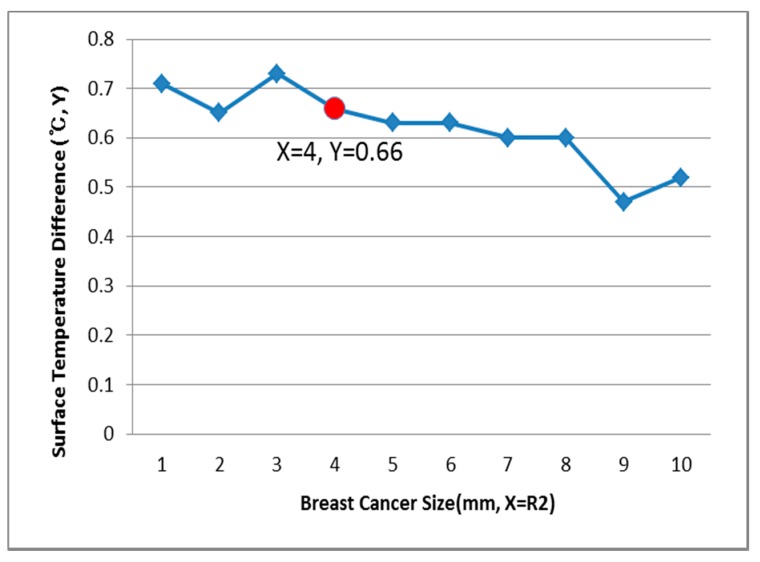
Surface temperature difference for R1 of 70 mm (X = 10, Y = 0).

**Figure 11:**
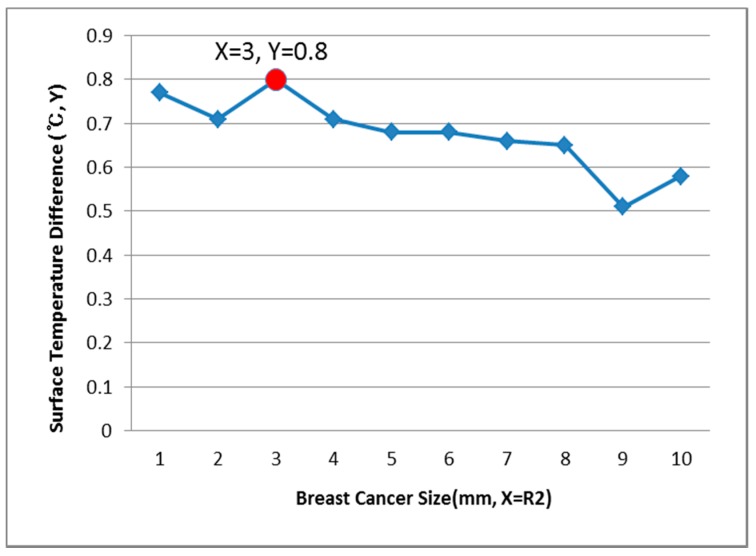
Surface temperature difference for R1 of 80 mm (X = 10, Y = 0).

Figure 9, Figure 10 and Figure 11 show the PDE thermal analysis, which was conducted at the breast cancer (defect) position X = 10, Y = 0, with breast test model sizes (R1) of 60, 70, and 80 mm. Figure 9, Figure 10 and Figure 11 provide a graphical means of displaying this analysis, assuming that this data is used in a medical setting.

In Figure 9, the breast cancer size and the difference in the change of the maximum and minimum of the breast test model surface temperature varied depending on the size and position. As an example, if R1 = 60 mm, then the temperature difference was 0.66 °C, and the breast cancer size was considered to be 1 mm. Figure 10 analyzes the data at R1 = 70 mm, and if the temperature difference was 0.66 °C, then the breast cancer size was 4 mm. Figure 11 analyzes the data at R1 = 80 mm, and if the temperature difference was 0.8 °C, then the breast cancer size was 3 mm [1,2,3,4].

**Figure 12:**
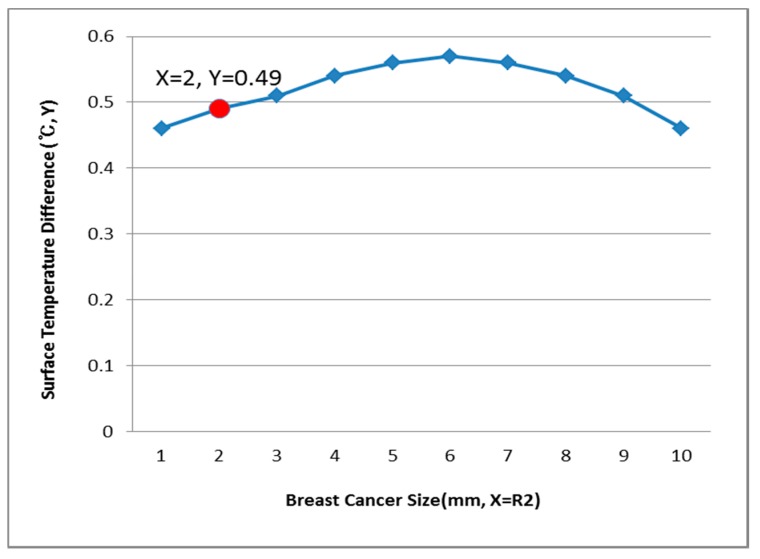
Surface temperature difference for an R1 of 60 mm (X = 10, Y = 10).

**Figure 13:**
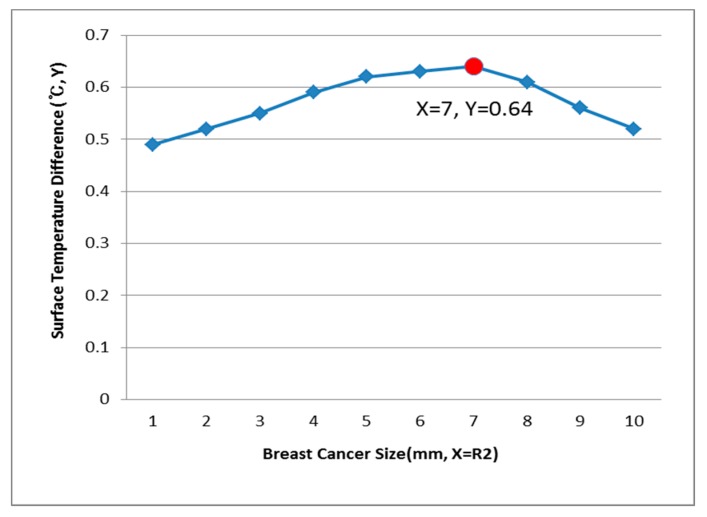
Surface temperature difference for an R1 of 70 mm (X = 10, Y = 10).

**Figure 14:**
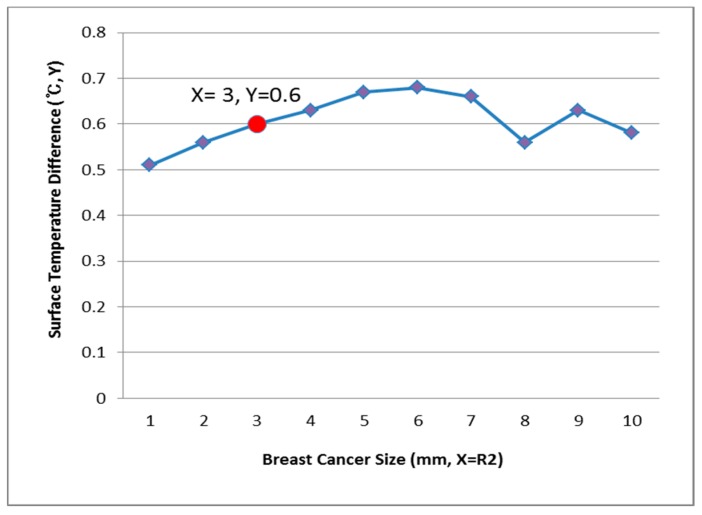
Surface temperature difference for an R1 of 80 mm (X = 10, Y = 10).

**Figure 15:**
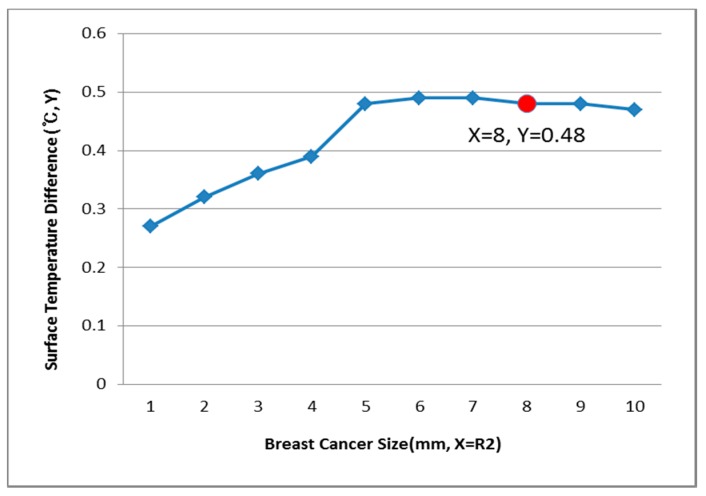
Surface temperature difference for an R1 of 60 mm (X = 0, Y = 10).

**Figure 16:**
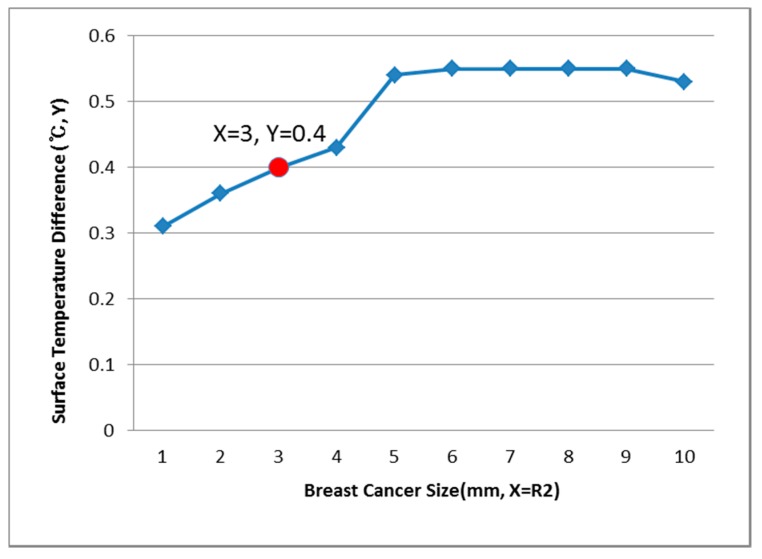
Surface temperature difference for an R1 of 70 mm (X = 0, Y = 10).

**Figure 17:**
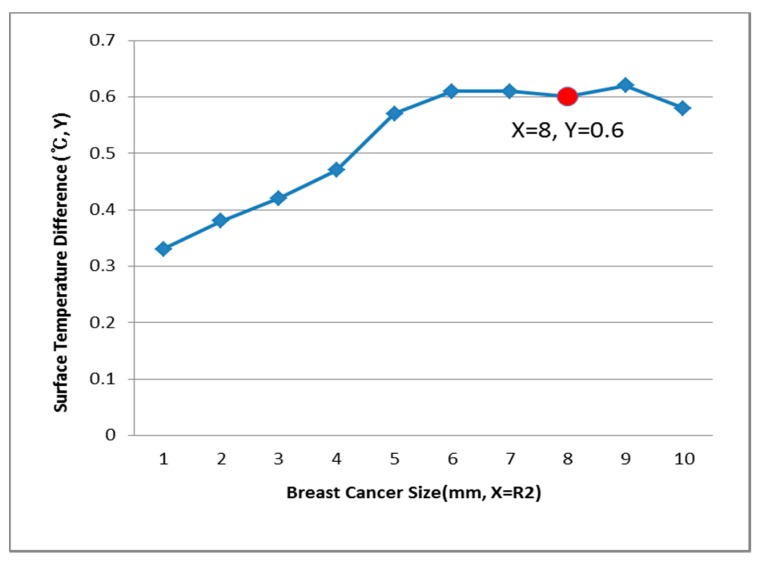
Surface temperature difference for an R1 of 80 mm (X = 0, Y = 10).

Figure 12, Figure 13 and Figure 14 have the same conditions at the breast cancer (defect) position X = 10, Y = 10. Figure 12 shows that when R1 = 60 mm and the temperature difference was 0.49 °C, the breast cancer size was 2 mm. Figure 13 shows that when R1 = 70 mm and the temperature difference was 0.64 °C, the breast cancer size was 7 mm. Figure 14 shows that when R1 = 80 mm and the temperature difference was 0.60 °C, the breast cancer size was 3 mm. Further, Figure 15, Figure 16 and Figure 17 show the results for breast cancer sizes of 8 mm, 3 mm, and 8 mm at the breast cancer position (X = 0, Y = 10).

Thus, if the bilateral symmetry of the breast cancer position was the same, the PDE thermal analysis was also the same.

The important points of this research are as follows.

1. If skin material and breast cancer data can be accumulated, the defects can be predicted without wounding the material.
2. By using pure heat, it is possible to harmlessly predict flaws in the human body.
3. This technique is applicable to building structures and various other materials, but it is necessary to construct a model using data for each material [23,24,25].
4. The prediction of breast cancer via thermal imaging and thermal analysis is a future research direction that should be explored further [26,27,28,29,30,31].
5. Main arteries and blood flow intensity are important agenda items for our next thermal analysis study.
6. In future studies, we should obtain the help of electronic engineers, and the digital work and the clinical experiment regarding the size of the breast cancer, which were observed immediately after the thermal analysis, should be conducted concurrently.

## 4. Conclusions

The surface temperature difference was analyzed using PDE analysis via two-dimensional modeling to predict breast cancer, and the contributions of this study are summarized as follows:

- It is possible to estimate the presence, position, and size of breast cancer in a two-dimensional model through PDE thermal analysis.
- Applied PDE analysis can be used to predict the presence, position, and size of breast cancer, specifically taking into account the surface temperature difference, and if the breast cancer position exhibits bilateral symmetry on the left- and right-hand sides, the analysis is the same.
- As the diameter of the breast model increased, heat transfer decreased, the surface temperature decreased, and the surface temperature difference increased generally.
- If the breast cancer (R2) position was (X = 10, Y = 0), the test model size (R1) was 50 mm, and the surface temperature difference was 0.60 °C, then the breast cancer at R2 was estimated as 3 mm, and the rest of the data were listed in the breast cancer estimation charts.
- If the relationship between the test material and the defect is varied and data are accumulated, then this model can be used for various different structures, including tumors and various cancers.

The diagnosis of breast cancer via theoretical investigations of defective specimens and two-dimensional PDE models confirmed the importance of improving such medical diagnoses and the necessity of expanding technology in various fields in the future.

## Figures and Tables

**Table 1:** Theoretical data for breast cancer analysis at various positions (X = 10, Y = 0).

	R1	40 mm	50 mm	60 mm	70 mm	80 mm
R2						
**1 mm**		0.50	0.58	0.66	0.71	0.77
**2 mm**		0.42	0.51	0.69	0.65	0.71
**3 mm**		0.52	0.60 ℃	0.67	0.73	0.80
**4 mm**		0.34	0.51	0.59	0.66	0.71
**5 mm**		0.48	0.50	0.57	0.63	0.68
**6 mm**		0.35	0.48	0.55	0.63	0.68
**7 mm**		0.40	0.46	0.53	0.60	0.66
**8 mm**		0.34	0.40	0.52	0.60	0.65
**9 mm**		0.47	0.32	0.41	0.47	0.51
**10 mm**		0.32	0.47	0.47	0.52	0.58

**Table 2:** Theoretical data for breast cancer analysis at various positions (X = 10, Y = 10).

	R1	40 mm	50 mm	60 mm	70 mm	80 mm
R2						
**1 mm**		0.35	0.42	0.46	0.49	0.51
**2 mm**		0.37	0.44	0.49	0.52	0.56
**3 mm**		0.40	0.46	0.51	0.55	0.60
**4 mm**		0.42	0.48	0.54 ℃	0.59	0.63
**5 mm**		0.43	0.52	0.56	0.62	0.67
**6 mm**		0.44	0.52	0.57	0.63	0.68
**7 mm**		0.41	0.50	0.56	0.64	0.66
**8 mm**		0.48	0.47	0.54	0.61	0.56
**9 mm**		0.31	0.48	0.51	0.56	0.63
**10 mm**		0.32	0.49	0.46	0.52	0.58

**Table 3:** Theoretical data for breast cancer analysis at various positions (X = 0, Y = 10).

	R1	40 mm	50 mm	60 mm	70 mm	80 mm
R2						
**1 mm**		0.22	0.25	0.27	0.31	0.33
**2 mm**		0.27	0.28	0.32	0.36	0.38
**3 mm**		0.23	0.31	0.36	0.40	0.42
**4 mm**		0.24	0.33	0.39	0.43	0.47
**5 mm**		0.29	0.41	0.48	0.54	0.57
**6 mm**		0.30	0.42	0.49	0.55 °C	0.61
**7 mm**		0.31	0.42	0.49	0.55	0.61
**8 mm**		0.35	0.49	0.48	0.55	0.60
**9 mm**		0.33	0.53	0.48	0.55	0.62
**10 mm**		0.32	0.48	0.47	0.53	0.58
